# Diagnostic accuracy of combinatorial mRNA biomarkers for non-invasive detection and therapy monitoring of oral and oropharyngeal SCC

**DOI:** 10.1038/s41416-025-03313-w

**Published:** 2026-01-08

**Authors:** Leonie Hose, Alina Celine Tekin, Bart Verwaaijen, Rayoung Kim, Christian Rückert-Reed, Ulrich Hamberger, Tobias Busche, Lars-Uwe Scholtz, Frank Brasch, Ingo Todt, Peter Goon, Matthias Schürmann

**Affiliations:** 1 Department of Otolaryngology, Head and Neck Surgery, Klinikum Bielefeld-Mitte, Medical School OWL, Bielefeld University, Bielefeld, Germany; 2 Core Unit Laboratory Research at Department of Laboratory Medicine, Microbiology and Transfusion Medicine, Klinikum Bielefeld— Mitte, Bielefeld, Germany; 3 Medical School OWL, Bielefeld University, Bielefeld, Germany; 4 Center for Biotechnology (CeBiTec), Bielefeld University, Bielefeld, Germany; 5 Department of Pathology, Klinikum Bielefeld—Mitte, Bielefeld, Germany; 6 Department of Medicine, Yong Loo Lin School of Medicine, National University of Singapore and National University Health System, Singapore, Singapore

**Keywords:** Oral cancer detection, Diagnostic markers

## Abstract

**Background:**

Oral squamous cell carcinoma (OSCC) is increasingly common, with over 380,000 new cases annually. Despite its high incidence (6.0 per 100,000 males; 2.3 per 100,000 females) and poor prognosis, no molecular biomarkers exist for early detection. Non-invasive sampling could improve diagnosis and patient outcomes.

**Methods:**

This pilot study used RNA sequencing to identify significantly upregulated mRNA targets in swab samples from oral and oropharyngeal SCC patients and healthy probands. After filtering, four potential biomarkers were further validated in 79 samples using RT-qPCR. CombiROC analysis assessed diagnostic performance. Additional RT-qPCR on tumour and normal tissues and fluorescence staining in FFPE tumour sections evaluated expression at mRNA and protein levels.

**Results:**

A panel of three markers (c-JUN, SFN, HSP90AB1) showed high diagnostic accuracy: 92.3% specificity, 92.3% sensitivity, and AUC of 0.91. Fluorescence staining confirmed significantly higher protein expression in tumour tissues, supporting RNA findings. The panel showed stronger diagnostic performance in men than in women.

**Conclusion:**

This study presents a promising non-invasive biomarker panel for oral and oropharyngeal SCC detection. Further validation in larger cohorts is needed to confirm diagnostic value and clarify sex specificity. The approach is adaptable to other tumour types and sample materials, supporting molecular diagnostics.

**Figure:**
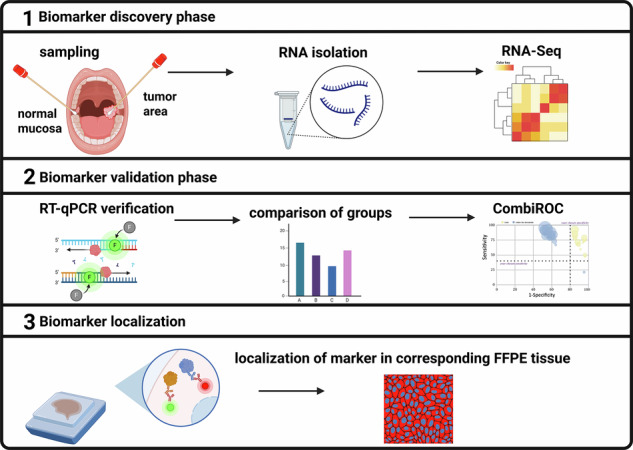
The workflow shows all project steps from sampling to marker localisation in FFPE tissue. In the discovery phase, RNA sequencing was used to find potential mRNA markers in swab samples. Via strict filtering, markers for RT-qPCR were selected and verified in a bigger cohort in the validation phase. Selected markers were then used to do combinatorial analysis to improve the specificity and sensitivity compared to that of the single markers. Finally, markers were verified and localised in primary oral and oropharyngeal SCC FFPE tissue. Created with BioRender.com.

## Background

Head and neck tumours, particularly oral squamous cell carcinomas (OSCC), represent a significant health challenge worldwide and remain a highly dynamic, multifaceted disease with global significance. OSCC accounts for over 90% of all malignancies within the oral cavity, with an annual incidence exceeding approximately 390,000 new cases [1] globally. The incidence and prognosis of OSCC show notable sex-related differences. Epidemiological studies consistently report a higher prevalence of OSCC in males compared to females, with a male-to-female ratio of for example 2.2:1 global and 2.7:1 in Germany [2]. Key risk factors include tobacco and alcohol consumption, and human papillomavirus (HPV) infection. Especially for oropharyngeal SCC HPV infection plays an important role, e.g. HPV high-risk strains, like HPV16 and HPV18, have been identified as substantial risk factors for the younger population [3, 4]. For SCC of the tongue and tonsils, traditionally p16 immunohistochemistry is used as a reliable surrogate marker for HPV determination [5]. Tobacco, in both combustible and non-combustible forms, is strongly associated with OSCC, as it contains carcinogenic compounds that damage the oral mucosa[6, 7]. Other factors include poor oral hygiene and dietary deficiencies, which can exacerbate the risk of OSCC [8]. Oral and oropharyngeal SCC are often diagnosed at a late stage, necessitating major surgical interventions in critical regions of the mouth and throat, and these can have enormous consequences for a patient’s quality of life [9]. Thus, timely and accurate diagnostic techniques are essential in improving patient outcomes in the future. The current diagnostic gold standard for OSCC involves tissue biopsies followed by histopathological evaluation. Sampling tissue biopsies is invasive, anaesthetics must be administered, and it can result in patient discomfort, delays in treatment, and potential complications such as infection. In addition, endoscopy for screening is much more cost-intensive than e.g. PCR procedures. Biomarker research is therefore of great importance for diagnostics, and conducting it in minimally invasive material could be of great benefit not only to patients but also to medical staff. In the USA, for example, PCR tests cost between $40 and $200 and therefore offer a suitable alternative method in terms of cost benefit. In recent years, there has been a growing interest in non-invasive diagnostic methods, for example liquid biopsy like saliva, and cytology-based with swab samples, that could provide equally reliable results without the associated risks and avoid sampling by a clinician [10]. Besides that, molecular biomarkers have an increasingly significant in the diagnosis and prognosis of various cancers. Biomarkers such as p53 mutations, cyclin D1 overexpression, and alterations in EGFR (epidermal growth factor receptor) signalling pathways have been extensively studied in OSCC [13] and play an important role for prognosis and pathogenesis [11–13] and are used to assess the success of treatment and prognosis, but not for diagnostics in non-invasive material so far. Biomarkers that have been approved for other cancers and are already used to aid diagnosis or monitor progression include PSA for prostate cancer [14], AFP for hepatocellular carcinoma [15], and CA-125 for ovarian cancer [16].The detection of circulating tumour DNA (ct-DNA) and microRNAs (miRNAs) in saliva and plasma has shown significant potential for early-stage detection in recent years [17]. Additionally, testing for circulating cell-free HPV DNA (cf-HPV DNA) has been analysed as a minimally invasive alternative for diagnosis and monitoring of oral and oropharyngeal SCC. Despite this promising approach, current cf-DNA-based methods have limitations, such as tumour load-dependent sensitivity, cf-DNA fragmentation/degradation and interpretation difficulties, and have not yet been clinically standardised [18–21]. Furthermore, the regulation of mRNA in tumours plays a crucial role in cancer biology, influencing gene expression and tumour progression. mRNA expression signatures can serve as biomarkers for diagnosis, prognosis, and therapeutic response. For example, post-transcriptional modifications can affect the expression level of specific transcripts in tumour cells significantly [22]. Research is therefore already being conducted in various studies on mRNA markers for OSCC in tissue [23, 24] as well as in blood and saliva [25, 26], but so far, there are no approved clinical biomarkers. RT-qPCR is a precise, sensitive, and cost-effective tool for cancer diagnostics, offering quantitative detection of tumour-associated gene expression [27–30]. It holds much promise for future cancer screening, particularly with non-invasive samples, as a supportive alternative to traditional biopsies. In this study, our target was to identify potential mRNA markers in swab brush material of patients with oral and oropharyngeal SCC, for less invasive diagnostic and therapy control options.

## Materials and methods

### Participant consent and study design

This study was approved by the Ethics Committee of the university hospital Ruhr-Universität Bochum in Bad Oeynhausen, Germany (2022_060_1). All participants gave written informed consent according to the agreed patient information sheets. All methods were carried out in accordance with relevant guidelines and regulations. This was a prospective diagnostic accuracy study. Therefore, this manuscript was written in accordance with the STARD (2015) guidelines for diagnostic accuracy studies [31] and in part on the TRIPOD checklist, particularly with regard to the description of data collection, and the presentation of diagnostic performance indicators, to ensure the transparency of the study [32]. Patients were consecutively enrolled at the Department of Otolaryngology, Head and Neck Surgery, University Hospital OWL, during the period from November 2022 to November 2024. Eligible participants underwent non-invasive sample collection for RT-qPCR testing of predefined mRNA biomarkers (discovery phase). The histopathological diagnosis served as the reference standard. The mRNA biomarker panel was identified in a prior discovery phase using sequencing in a separate small cohort. The study overview is shown transparently in a flow chart in Supplementary Fig. 1. The sample size was based on practical constraints and informed by comparable studies that reported similar effect sizes with equivalent sample sizes. It was considered methodologically appropriate given the study’s design and scope.

### Cohort subgroups

Different cohort groups were defined as follows. Healthy subjects were people without abnormalities or symptoms in the oral/oropharyngeal area. Tumour patients were patients with acute oral or oropharyngeal SCC, who had not yet received treatment (initial diagnosis). The post-therapy group consisted of patients who had an oral/oropharyngeal SCC, had undergone treatment (e.g. surgery/radio chemotherapy) and were monitored regularly in the further course. We defined the high-risk group as patients presenting with symptoms such as swallowing difficulties, sore throat, dysphagia, or an oral foreign body sensation, in whom SCC had not been confirmed histologically. Despite the absence of histopathological evidence of malignancy, these patients were considered at potential risk due to the persistence and nature of their symptoms. All tumour-suspected diagnoses were confirmed or revised by biopsies.

### Inclusion/exclusion criteria

Included were men and women between 18 and 85 years of age. Patients for planned endoscopy with symptoms such as sore throat, foreign body sensation, dysphagia, dyspnoea with/without risks such as tobacco/alcohol consumption, complaints with/without suspected tumour. Exclusion criteria were pregnant women and patients with other additional different/previous tumour diseases.

All relevant clinicopathological and lifestyle factors are summarised in Tables 1 and 2.

**Table 1:** Clinicopathological and lifestyle information of all included participants.

		Healthy	Tumour	Post-therapy	High risk
Parameter	Category	Number *n*	Number *n*	Number *n*	Number *n*
Sex	Female	19	11	-	-
	Male	12	14	12	11
	Total *N*	31	25	12	11
Age	Mean	46	65	66	60
	Min	20	39	53	41
	Max	82	85	82	80
Cigarettes/day	0	21	8	8	4
	10–20	1	1	-	4
	21–30	3	0	-	2
	31–60	1	8	1	-
	NA	5	8	3	1
Alcohol	No alcohol	16	6	3	8
	Occasional	13	6	1	2
	Daily	2	8	4	1
	NA	-	5	4	-

**Table 2:** Clinicopathological information of male tumour patients and post-therapy patients.

	Category	ICD-code	Tumour	Post-therapy
			Number *n*	Number *n*
Localisation of primary tumour	Oropharynx	C09.0	7	8
		C10.9		
		C10.1		
	Floor of mouth/oral cavity	C04.9	1	-
	Tongue	C02.9	6	4
		C02.1		
		C10.4		
Primary tumour staging	T1		3	4
	T2		5	5
	T3		1	2
	T4		5	1
Nodal staging	N0		5	4
	N1		5	3
	N2		1	5
	N3		2	-
	NA		1	-
Metastasis staging	M0		11	11
	M1		-	-
	NA		3	1
Grading	G1		3	1
	G2		3	3
	G1-G2		3	-
	G3		-	1
HPV detection by p16 surrogate marker	p16 neg.		10	7
	p16 pos.		4	5
Therapy	Radiochemotherapy + Tumour resection + Neck dissection		7	6
	Tumour resection		3	4
	Neck dissection			
	Radiochemotherapy		1	1
	NA		3	1

### Cytology staining and microscopy

Cytological preparations for swab samples were rolled directly on a slide and fixed with M-FIX spray (Sigma Aldrich, Darmstadt, Germany). After this, slides were stained using Papanicolaou´s stain at the Department of Pathology, Klinikum Bielefeld. Light microscopy was performed by a Pathologist at a ×200 magnification.

### Sampling and sample preparation

Before professional swab brush tests were carried out by a physician, all patients got a 0.9% NaCL mouthwash solution, to rinse their mouth for 20 s.

Samples were taken as shown in Table 3.

**Table 3:** Sampling scheme of the smear test for the different test groups.

	Swab 1 test	Swab 2 control
Healthy group	-	Normal mucosa (Tonsil/Cheeks)
High-risk group	Conspicuous region	Normal mucosa (Tonsil/Cheeks)
Tumour group	Conspicuous region	Normal mucosa (Tonsil/Cheeks)
Post-therapy group	Ex- conspicuous region	Normal mucosa (Tonsil/Cheeks)

For tumour patients, the tumour area was swabbed directly (Swab1) with an Orcellex^®^ Brush (Rovers Medical Devices, Netherlands), as well as an area with macroscopically inconspicuous mucosa (Swab2). Patients with conspicuous regions were also swabbed directly at the affected sites (Swab1) and normal mucosa (Swab2), while healthy probands had their tonsils and cheeks swabbed (Swab1). The swab was then placed in an Eppendorf tube filled with 300 µl 1×RNA Shield (Zymo Research, Europe GmbH) for protection. The samples were cooled on ice until sample preparation proceeds. In the laboratory, the samples were shaken for at least 30 min at 900 rpm to release the cells from the swab. The brush was then removed and the tube was frozen at −80 °C until the RNA isolation.

### RNA isolation

RNA isolation was performed using the Quick-RNA Microprep Kit from ZymoResearch and was carried out according to the manufacturer's instructions.

### cDNA synthesis

cDNA synthesis was performed with a cDNA synthesis kit (all priming options) from Biozym Scientific GmbH. The synthesis is carried out with random hexamer primers and was performed according to the manufacturer’s instructions. After synthesis, all samples were diluted 1:50 with water.

### RNA sequencing

The following samples were selected for RNA-Sequencing: Tumour *n* = 4, healthy smoker *n* = 3, healthy non-smoker *n* = 3. After RNA isolation and quality control, poly (A)-selected libraries were prepared of total RNA using QuantSeq 3′mRNA-Seq Library Prep Kit FWD for Illumina (Lexogen), according to manufacturer’s instructions. Size distribution and quality of the libraries were assessed by Tape Station Analysis Software 4.1.1 (Agilent Technologies) and final libraries were sequenced 75 bp single-end mode on a NextSeq2000 with a 3 chemistry. Raw sequence reads were filtered and trimmed with fastp. The output was mapped with Hisat2 (https://www.nature.com/articles/s41587-019-0201-4, version 0.19.11) against the GCF_000001405.40_GRCh38.p14 genomic reference. The resulting sam files where converted to bam, sorted and indexed with samtools (https://academic.oup.com/gigascience/article/10/2/giab008/6137722?login=false, version 1.8). Counts where assigned to genomic features with featureCounts (https://academic.oup.com/bioinformatics/article/30/7/923/232889, version 1.4.6).

### Primer design

For RT-qPCR analysis, different gene candidates were chosen. Sequences of the targets were selected with Ensemble Genome Browser (Ensembl genome browser 111). Primers were designed and verified with the NCBI Primer Tool (Primer designing tool (nih.gov)). All primers are listed in Table 4, with corresponding sequences. For normalising the housekeeping gene *MT-ATP6*, was used.

**Table 4:** All primers described in this work including forward and reverse sequence.

Gene symbol	Forward 5′-3′	Reverse 3′-5′
MT-ATP6	TTCATTGCCCCCACAATCCT	TGGGGATCAATAGAGGGGGA
c-JUN	TTCACCTTCTCTCTAACTGCCC	TCTGGACACTCCCGAAACAC
SFN	TAGCCTATAAGAACGTGGTGGG	CCTCGTTGCTTTTCTGCTCAAT
HSP90AB1	TGGTAGACACAGGCATTGGC	CCAGACTTGGCAATGGTTCC
STARD7	CTCCCTGCCTGCACAATATCA	AACTGGCCGATTCACAGGAAA
TRIMM	GGGAGTGTGTCCTTTGAGCA	AGAATTGGGGTTCTCCCACG
RRAD	TGGGTTAGAGGTCTGGAGT	AACACACAACACATCTGC
LCE2C	GACTGCTGTGAGAGTGAACCT	TTGGCCATTCAGTCCCAAGA
MYOB1	TAGCATGGGTCAGAGTGGGA	GCAGCCTCAGCTTCCATACA
EIF5B	AGCGAAGAATTGGAAGATAAAGATT	TCACTCCCAGAGTACATTTCCAC

### RT-qPCR

For all RT-qPCR reactions, a ready-to-use master mix (Luna Universal qPCR Mix, New England Biolabs) was used. All measurements were run in triplicates, with a total volume of 10 µl each. The analyses were carried out with the MIC cycler from BioMolecular Systems (Hessisch Oldendorf, Germany).

### Histopathology of FFPE Tissue

Paraffin-embedded tissue was sectioned to 2 µm thickness using a sliding HM430 microtome (Zeiss). Hematoxylin/Eosin (HE) staining was performed using standard protocols in a linear COT 20 tissue stainer (MEDITE, Burgdorf, Germany).

### Indirect immunofluorescence (IF) of FFPE Tissue

For IF analysis, paraffin-embedded tissue was sectioned to 3 µm thickness. FFPE tissue was dewaxed in a descending alcohol series and then unmasked in 10 mmol citric acid/ Na citrate buffer, followed by blocking in PBS + bovine serum albumin (BSA) 0.1% for 15 min. DNA was stained with DAPI (Sigma Aldrich, St. Louis, MO, United States) using 1 mg/ml 1:500 diluted in PBS + BSA 0.1% (Capricorn Scientific). The following primary antibodies were used: Anti-c-JUN (mouse monoclonal; 1:100; BD Biosciences), anti-HSP90AB1 (mouse monoclonal; 1:100; Santa Cruz Biotechnology, Heidelberg, Germany), anti-SFN (mouse monoclonal; 1:100; Santa Cruz Biotechnology, Heidelberg, Germany). Tissue sections were washed for 10 min with PBS + 0.1% BSA and subsequently incubated with species specific secondary antibodies (Alexa 555 conjugated; anti-mouse; 1:400) for 2 h. Finally, tissue sections were washed 10 min in PBS + 0.1% BSA and 5 min in distilled water. After complete drying they were covered with mounting medium (Thermo Scientific, Rockford, U.S.A). Images were created using Keyence BZ-X800 software, and ImageJ.

### Statistical analysis

Statistical analysis of the RNA sequencing data has been done in R version 4.3.3. and with DEseq2 version 1.42.1. Further processing and visualisation were done with the Tidyverse version 2.0.0, version 3.70.0 and ggplot2 version 3.5.1 packages. All RT-qPCR analyses were performed with Graph Pad version 8.0.2. Two-tailed Welch’s *t* tests were utilised for determining significant differences. A *p*-value less than 0.05 is considered statistically significant. To evaluate the diagnostic accuracy of selected genes, receiver operating characteristics (ROC) were performed with Graph Pad version 8.0.2. Additionally, the R-based tool CombiROC was used to analyse diagnostic values of combined genes and cut off values of single markers and marker combination by maximising the Youden index [33].

## Results

**Fig. 1:**
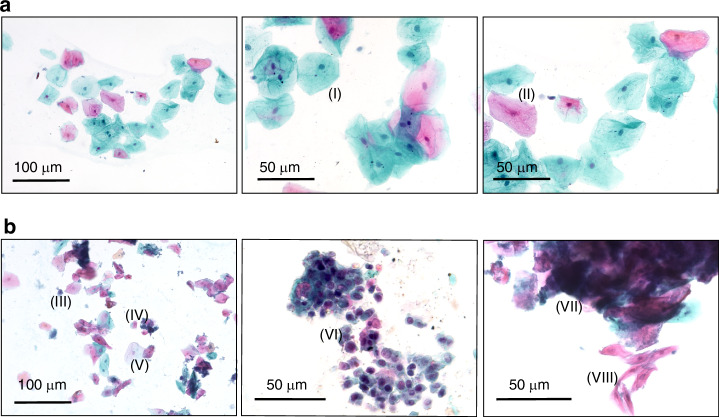
Source material for molecular analysis. Representative images of PAP-staining from normal buccal smear from a healthy control in (**a**) and an OSCC patient in (**b**). (**I**) normal intermediate cell, (**II**) normal superficial cell, (**III**) bacteria aggregate, (**IV**) atypical cells, (**V**) normal intermediate cell, (**VI**) poor-differentiated tumour cells, (**VII**) 3-dimensional cell cluster, (**VIII**) spindle shaped squamous cancer cells. Scale bars: 100 µm and 50 µm. Smears of the tumour patients contain tumour cells that are required for further molecular analysis.

As described above, swab samples were taken from tumour patients as well as from different control groups. These were visualised by Papanicolaou staining, and representative images are shown in Fig. 1. Figure 1a illustrates a smear of healthy oral mucosal cells. Blue intermediate cells (I) and typical red-stained superficial cells (II) were found and show loose aggregations. The nuclear-cytoplasm ratio is regular, and the slide background appears clean. Figure 1b highlights a disordered cell image with cell clusters and dirty background with various leucocytes and bacteria (III). Atypical/dysplastic (IV) cells can already be recognised between the healthy mucosal cells (V), poorly-differentiated tumour cells with irregular nuclear-cytoplasm-ratio, basophilic cytoplasm, hyperchromasia and visible nuclei are shown (VI). A big 3-dimensional cell cluster is shown on the right (VII) and (VIII) provided more differentiated squamous cancer cells, with long spindle shapes and eosinophilic cytoplasm, as well as nuclear pleomorphism. Detection and collection of tumour cells like (VI) and (VIII) with our swabs are necessary for accurate molecular biological analyses.

### Biomarker discovery phase

**Fig. 2:**
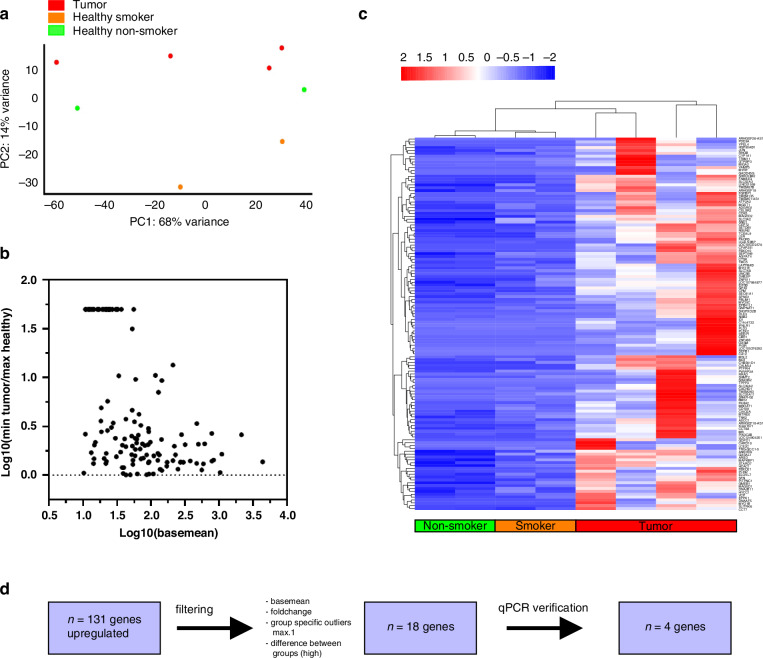
Sequencing data analysis and biomarker discovery. **a** PCA analysis showing clustering of the different grouping component 1 with 68% and component 2 with 14%. **b** Dotplot of *n* = 131 genes with main filtering step shows the basemean against ratio between min Tumour patient and max Healthy proband (smoker and non-smoker). These genes were shown additionally in the heatmap in **c** DEGs hierarchical evaluation for clustering in healthy and tumour samples. **d** Shows the way of filtering from *n* = 131 genes to *n* = 4 genes.

In the discovery phase of this pilot study, RNA sequencing was used to profile differential gene expression of *n* = 4 male tumour patients, *n* = 3 male healthy non-smoker and *n* = 3 male healthy smoker. For each of the replicates between 10762608 and 41752923 reads were sequenced of these, between 443099 and 20105974 could be assigned to gene coding regions after processing. One sample from the group of healthy non-smokers and one sample from the group of healthy smokers were excluded from further analyses because of deviations that were found between the technical replicates and reduced amounts of assigned reads. For all downstream analyses, the technical replicates were combined. RNA-Sequencing data are shown in Fig. 2. PCA analysis was used to assess the variances in gene expression levels between healthy probands and tumour patients. The first component 1 (PC1) accounted for 68% of the overall variance of the data, and component 2 (PC2) accounted for 14%. As shown in Fig. 2a, the control samples and the tumour samples were separated, a distinction can also be made between healthy smokers and non-smokers.

### Differentially expressed genes

RNA-Sequencing results in the detection of 23,114 different transcripts among the 8 samples. Of these, 2486 were found in all the samples, whereas each individual sample had detectable transcript levels for between 6592 and 12,181 protein coding genes. Sequencing results are summarised shown in Fig. 2. In the tumour samples, 903 transcripts are significantly differentially abundant compared to smokers and 799 transcripts compared to non-smokers by a fold change (FC) greater than or equal to 2 and with a Padj of less than 0.05. Figure 2b shows over expressed genes (*n* = 131) that have significance (*P*_adj_ < 0.05) between all healthy and all tumour samples (independent of smoking status). We calculated the largest minimum Fold Change from our RNA-seq experiment to maximise the chance of obtaining biomarkers that are robust enough for RT-qPCR. Therefore, we took the DEGs identified by DESeq and calculated the fold change by matching the highest count from condition one with the lowest count from condition two:${\mathrm{Largest}}\,{\mathrm{Minimum}}\,{\mathrm{Fold}}\,{\mathrm{Chang}}e=\max (\,\frac{\max \left({\mathrm{Healthy}}{\mathrm{Counts}}\right)}{\min \left({\mathrm{Tumor}}{\mathrm{Counts}}\right)})$. This approach was used to find transcripts with differences in abundance that were always detectable within the dynamic range of this experiment, independent of the patient. This is in contrast to conventional differential analysis, which instead displays biological significance when looking at the experiment as a whole, ignoring the possibility of outliers as long as the p-value is within range. The latter makes finding suitable biomarker candidates in a small cohort experiment very challenging. The heatmap (Fig. 2c) shows the clustering of all tumour patients, for these genes heathy smokers and non-smokers were clustered. Further filtering set up is presented under the heatmap. *N* = 131 genes are filtered through base mean > 25, log_2_FC > 2, difference between tumour/healthy baseman min. 50 and max. 3500. Additionally, further analyses were only carried out if there was $\le$ 1 outlier per tumour/control group for the respective target gene. This filtering strategy resulted in a reduction to *n* = 18 genes, which were then tested with RT-qPCR to verify whether they can be extrapolated to the entire cohort.

### Biomarker validation phase and tumour specificity

**Fig. 3:**
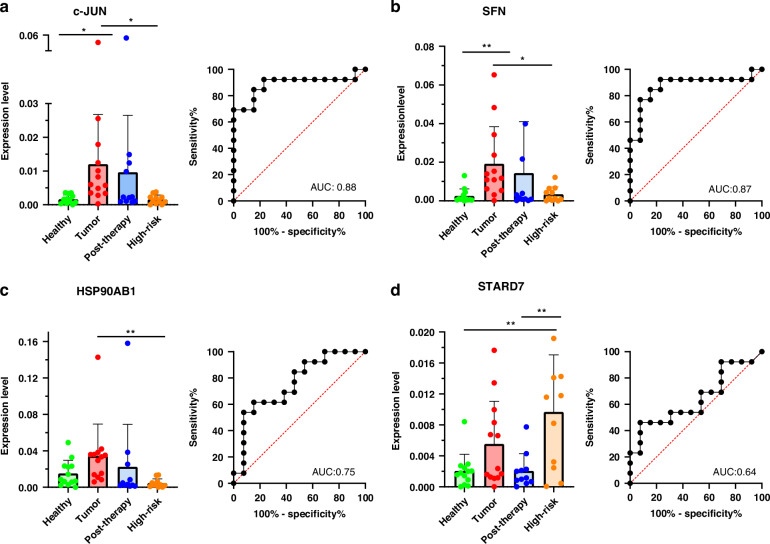
RT-qPCR biomarker validation and diagnostic performance of biomarker. Expression levels of all patient groups with markers **a** c-JUN, **b** SFN, **c** HSP90AB1 and **d** STARD7 are shown. The ROC curves give information about sensitivity and specificity of Healthy vs. Tumour for all markers. (Welch's *t* test, two-tailed, 95% confidence interval, *p < 0.05, **p < 0.01). Of all four markers, c-JUN shows the highest diagnostic precision for the discrimination of tumour patients and healthy probands, while STARD7 gives the worse AUC and highest expression in high-risk group.

After gene filtering as described in Fig. 2, RT-qPCR verification was done with *n* = 18 biomarkers. Most of these biomarkers showed different FC and expression levels in RT-qPCR analysis than expected and when considered to the Seq-Data (Supplementary Fig. 3). *N* = 4 genes showed expected differential gene expression RT-qPCR values and were further analysed. For *c-JUN, SFN, HSP90AB1* and *STARD7* RT-qPCR results, including tumour, healthy, post-therapy and high-risk samples were compared in Fig. 3. To investigate the diagnostic precision of these genes, we performed ROC analysis for every single marker. As shown in Fig. 3, the AUC was 0.88 for *c-JUN*, 0.75 for *HSP90AB1*, 0.87 for *SFN* and 0.64 for *STARD7*. Significantly higher expressions in tumour patients between healthy controls were measured for *c-JUN* (*p* = 0.0262) and *SFN* (*p* = 0.0090). The expression level between tumour and high-risk group is also significantly higher for *c-JUN* (*p* = 0.0257), *SFN* (*p* = 0.0126) and *HSP90AB1* (*p* = 0.0096). *HSP90AB1* shows a higher but not significant expression in tumour patients (*p* = 0.4922), compared to healthy controls as well as *STARD7* (*p* = 0.0509). While for *c-JUN, SFN* and *HSP90AB1* the expressions of the high-risk group are on average similar to those of the healthy group (indicate standard deviation), *STARD7* shows a significantly higher expression for this group (*p* = 0.0066). This differs only by 0.004 from the expression in tumour patients. Additionally, for *STARD7* is the only marker with significant differences between the expression in post-therapy and high-risk patients (*p* = 0.0067). All AUC, SE and SP values of these 4 markers between tumour and control groups are listed in Supplementary Table 1.

**Fig. 4:**
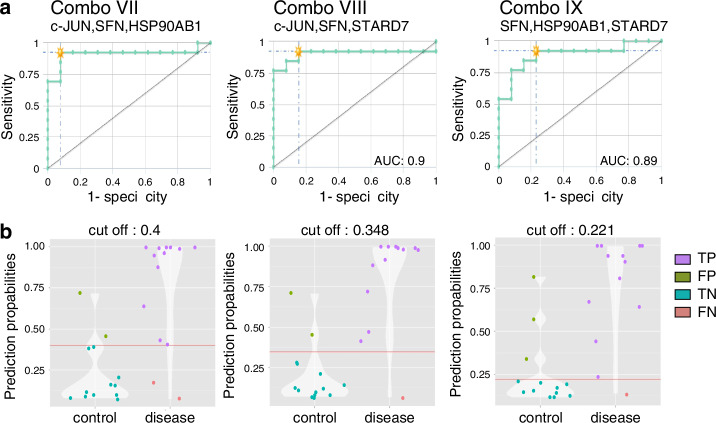
Best signatures to differentiate between tumour patients and healthy probands. Three best marker combinations for distinction between tumour patients and healthy probands are shown. **a** ROC curves show the diagnostic accuracy of the combinations. **b** Corresponding violin plots with cut off (red line). True positive values are provided in magenta, false negatives in green, true positives in turquoise and false negatives in red. The best marker combination to discriminate between tumour and healthy group is Combo VII and contains of c-JUN, SFN, HSP90AB1.

To improve the diagnostic accuracy, combinatorial analysis of these four markers was carried out with the tool CombiROC, as shown in Fig. 4. The combinatorial analysis achieved an AUC value of 0.91 through the combination of *c-JUN, SFN* and *HSP90AB1 (Combo VII)*, with a sensitivity (SE) and specificity (SP) of 92.3%. The cut-off value, that was given by the software, is 0.4 and resulted in 3.9% false positives and false negatives. Two additional combinations, which also improved the diagnostic accuracy of single markers to discriminate healthy from tumour samples (*Combo VIII and Combo IX*), are shown. *Combo VIII* provides an AUC value of 0.9, and *Combo IX* an AUC of 0.89, but had worse overall sensitivity values of 76.9% for *Combo VIII* and 84.6% for *Combo IX*. The best combination to distinguish between tumour and post-therapy patients consisted of *HSP90AB1* and *SFN* and achieved an AUC of 0.79 with SE 92.3% and SP 72.7%. An AUC of 1 was achieved with four different combinations, for differentiating between tumour and high-risk patients, which represented the best diagnostic precision (Supplementary Fig. 4). All AUC, SE and SP values of the combinations comparing the tumour with the different groups (*Combo I–XI*) are listed in Supplementary Table 2. Additionally, cut-off values of single markers are shown in Supplementary Table 3.

The male tumour cohort from Fig. 2 was divided into additional groups: HPV infection, tumour location and tumour stage, in order to measure a possible correlation between the biomarkers and the clinical data. However, no significant correlation was found between the expression of the markers and these factors (Supplementary Fig. 6). In the next step, we validated the results of the previously all-male cohort with a female cohort of *n* = 22 healthy and *n* = 10 tumour patients and established a comparison. In the female OSCC patients, an overall poorer discrimination between tumour and healthy individuals was measured in all biomarkers presented. The AUC in the female cohort was 0.69 for *c-JUN*, 0.54 for *HSP90AB1*, and 0.53 for *SFN*. This resulted in AUC differences of 0.2 for *c-JUN*, 0.3 for *SFN* and 0.2 for *HSP90AB1* between males and females. The RT-qPCR results and the corresponding ROC curves are shown in Supplementary Fig. 5.

### Biomarker localisation in tissue

**Fig. 5:**
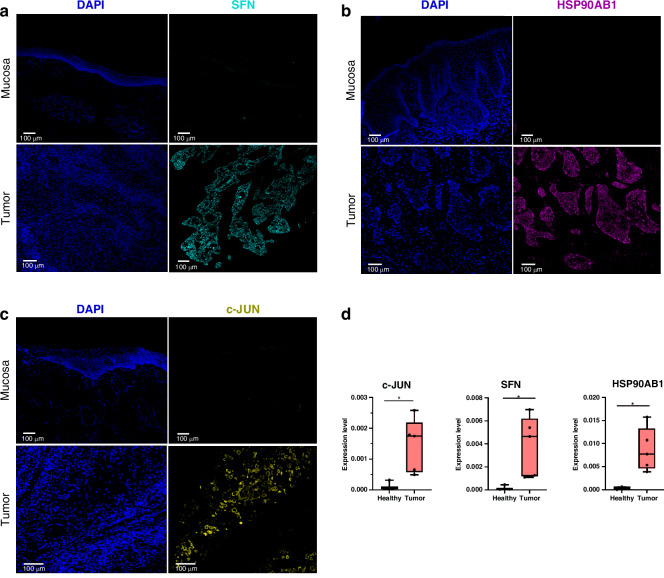
Immunofluorescence staining shows high expression in tumour tissue. **a** Expression of SFN (cyan) in healthy mucosa and tumour tissue. **b** Expression of HSP90AB1 (pink) in healthy mucosa and tumour tissue. **c** Expression of c-JUN (yellow) in healthy mucosa and tumour tissue. Scale bar = 100 µm. **d** mRNA expression level of SFN, HSP90AB1 and c-JUN in fresh tumour tissue (Welch's *t* test, two-tailed, 95% confidence interval, *p < 0.05, **p < 0.01). All markers show tumour-specific, upregulated expression compared to the healthy mucosa.

For biomarker characterisation and localisation, immunofluorescence staining was performed with antibodies against the proteins c-JUN, HSP90AB1 and SFN. Archived FFPE tissues from primary oral tumours were used. Figure 5 shows representative images of healthy mucosal areas as well as tumour areas. Provided are differential expression patterns of SFN, HSP90AB1, and c-JUN between mucosal and tumour tissues. Figure 5a shows that SFN (cyan) is minimally expressed in the healthy epithelium but highly upregulated in the tumour tissue, as evidenced by increased red fluorescence intensity. In Fig. 5b, HSP90AB1 (magenta) exhibits a distinct upregulation in tumour tissue compared to healthy tissue, where its expression is nearly absent. Figure 5c demonstrates c-JUN (yellow) expression, which, like SFN and HSP90AB1, is low in the healthy mucosa but prominently increased in the tumour tissue. For c-JUN, in addition to cytoplasmic staining, nuclear staining is also seen, indicating activation of c-JUN. While SFN and c-JUN clearly distinguish tumour cell clusters from stroma and surrounding tissue, we could also detect an increased expression of HSP90AB1 in the infiltrated lymphocytes in highly immune-infiltrated tumour areas (Supplementary Fig. 7). In addition, we performed RT-qPCR on homogenised fresh tissues from male patients to detect the expression of the markers in the tissue. These give significant differences between healthy tissue (*n* = 3) and tumour tissue (*n* = 5) (Fig. 5d). For *SFN*, the mRNA expression level in the fresh tumour tissue is about 15-fold higher (*p* = 0.0335), for *HSP90AB1* about 40-fold higher (*p* = 0.0167) and for *c-JUN* about 13-fold higher (*p* = 0.0263) than in healthy tissue.

## Discussion

### Exfoliative cytology for oral and oropharyngeal SCC diagnosis

In this study, we investigate the feasibility of a roadmap to identify mRNA biomarkers for swab based non-invasive diagnostics for oral and oropharyngeal SCC. In clinical practice, swabs have already emerged as a valuable non-invasive sampling approach for cytological and molecular diagnostics, offering several advantages and disadvantages. One of the primary benefits of using swabs is their ease of collection, which minimises discomfort for patients and allows for rapid sampling in various settings, including outpatient clinics. Swab based exfoliative cytology relies on scraping cells from oral lesions and examining them microscopically, as provided in Fig. 1. This approach is simple and minimally invasive, but cytology often yields lower sensitivity due to morphological overlap between benign and malignant cells, which can lead to diagnostic inaccuracies and false negatives (Fig. 1b, (V)) [34, 35]. Particularly experienced and trained personnel are required, and the thorough examination of cytological preparations takes time and is therefore cost intensive as well [36]. With molecular approaches, specific RNA expression patterns can be measured, which are directly associated with cancerous changes. It has already been demonstrated that molecular markers can improve detection accuracy, offering earlier and more definitive insights into oral and oropharyngeal SCC onset than cytology alone [37].

### Cohort selections for RNA-Sequencing and sex specificity of potential biomarker

In our pilot-study, we assumed that an extensive homogenisation of the cohort of our pilot study would be promising in terms of marker identification, we focused on the men with oral and oropharyngeal SCC. Males constitute more OSCC cases than women and are therefore more available (70–80%), globally [38, 39]. Indeed, we were able to show that sex-specific differences in the biomarker search for oral and oropharyngeal SCC diagnostics play a role and should be considered. The extrapolation of described markers to female patients resulted in overall lower AUC values (Supplementary Fig. 4). This allowed us to show in our cohort, that male tumour patients have a stronger diagnostic significance. This discrepancy may certainly be due to the fact that only male samples were used in the initial discovery of markers by RNA-seq, so that the particularities of the female samples were not considered. There is evidence suggesting that gene regulation in tumours may differ between males and females, which could account for the observed differences. For example, in glioma research it has already been shown, that sex-specific pathways like Wnt signalling and immune-related processes reveal unique biological pathways associated with each sex, which could lead to more precise diagnostic markers and therapeutic approaches for male and female patients [40]. Additionally, the tumour microenvironment (TME) also differs significantly between sexes. Women’s TMEs often showing higher immune cell infiltration and immune checkpoint expression [41]. This indicates that sex-specific markers might be also relevant for diagnostic purposes, highlighting the potential need for sex-tailored approaches in cancer diagnostics. Using a single-cohort study may limit the generalisability of biomarkers due to potential biases in population genetics or environmental factors, which can influence gene expression profiles [42]. We think that RNA sequencing with a bigger cohort and more diverse samples would allow the efficient initial validation of the robustness of potential biomarkers and facilitate broad clinical applicability [43]. Because of this, we would suggest selecting a bigger cohort with more samples to obtain universally applicable oral and oropharyngeal SSC markers.

### RT-qPCR verification and analysis of biomarker

In this pilot study, we used RT-qPCR, which allows the detection of specific mRNA molecules with high specificity and sensitivity and is also cost-effective and simpler to implement in a diagnostic laboratory, compared to other molecular diagnostic methods—especially next-generation sequencing [43–45]. In order to check the transferability to RT-qPCR, we filtered the RNA-Seq through *n* = 18 transcripts. It became apparent that the reproducibility of the sequencing data was only possible for *n* = 4 genes out of 18 (Fig. 3).

Supplementary Fig. 3ā shows exemplary expressions of *MYOB1, LCE2C, TRIMM, RRAD* and *EIF5B* in healthy and tumour swabs. The sequencing results could not be reproduced for these genes, which could be due to the limited sequencing cohort. These results are summed up in Supplementary Fig. 2B by comparing the FC for these genes from RT-qPCR and RNA-Seq. The values of the two methods are most similar for *c-JUN*. For *SFN, HSP90AB1* and *STARD7*, the log_2_FC of RNA sequencing is about twice as high as for RT-qPCR. The largest discrepancy exists between *RRAD* and *LCE2C*. When conducting an RNA-seq with a larger sample size, it would be advantageous to calculate statistical analyses, such as the confidence interval or standard deviation for the targets, in order to be able to predict how good the transferability is to the entire cohort. This was not reasonable within the framework conditions of a pilot study.

Smoking behaviour can also cause changes in the gene expression of various genes. This can be seen in Supplementary Fig. 2, which shows the differentially expressed genes between smoking tumour patients and healthy smokers. Examples for this are the genes *BACE2* and *CLC4A-AS1*, which appear to be upregulated in tumour patients. However, the RT-qPCR analysis in Supplementary Fig. 2B indicates that the expression level is also increased in healthy smokers. Our observation is consistent with several studies describing detectable changes in DNA methylation associated with tobacco smoking, which have consequences for gene expression [46] and that some of these genes were linked to cancer development [47]. Logically, stopping smoking behaviour could also be measured in the gene expressions of nasal epithelia by downregulating certain genes [48]. This highlights the importance of selecting a suitable and specific cohort for biomarker identification, in which both clinical parameters and behavioural patterns, such as smoking, should be considered.

The comparison of different subgroups of our cohort is shown for these four genes in Fig. 3. *c-JUN* and *SFN* show the strongest expression differences in the tumour group compared to the healthy cohort. *C-JUN, SFN* and *HSP90AB1* show similar expressions for the high-risk group, which includes healthy smokers and patients with inflammatory diseases, as for the healthy control group. This indicates that these three genes are not upregulated by inflammatory processes and show good tumour specificity and could be more suitable for diagnostics, while this was limited for *STARD7*. *STARD7* behaves differently for the high-risk group and seems to be less tumour-specific. *STARD7* is already known for its lipid transport functions, and also plays important roles in inflammatory processes and cellular response mechanisms. It has been shown to impact phospholipid transport, especially phosphatidylcholine delivery to mitochondria, which is crucial for maintaining cellular energy balance and responding to metabolic demands in inflammation [49, 50]. Different studies have shown changes in *STARD7* mRNA levels in carcinoma [51, 52]. But also, in other pathological conditions like inflammation, metabolic disorders, and neurological diseases [53]. These findings highlight the protein’s involvement in a wide range of disease states and cellular processes, which limits its suitability as a targeted marker for cancer diagnostics.

*c-JUN* is a transcription factor encoded by the *c-JUN* proto-oncogene and forms together with c-FOS a component of the AP-1 (Activator Protein-1) complex [54]. It plays a critical role in cellular processes such as proliferation, apoptosis, and tumour progression. It regulates gene expression by binding to AP-1 sites in the promoters of target genes, influencing various oncogenic pathways. In HNSCC, c-JUN expression correlates with the aggressiveness of the tumour and a poor prognosis. High c-JUN activity is observed in HPV-negative HNSCC, often in association with TP53 mutations [55]. c-JUN involvement in epithelial-mesenchymal transition (EMT) enables metastasis [56]. JNK phosphorylation activates c-JUN, this pathway is commonly hyperactivated in HNSCC, partly due to mutations in upstream regulators like TP53 and overexpression of EGF. Crosstalk between JNK and PI3K/Akt pathways enhances tumour invasiveness and chemoresistance [57]. Interestingly, c-JUN expression has been shown to increase with oral dysplasia severity, and higher levels in lesions that progressed to carcinoma suggest its involvement in early carcinogenesis [58–60]. *SFN* plays a significant role in regulating cellular processes such as apoptosis, cell cycle progression, and signal transduction. In cancer, particularly in OSCC as well as in pancreatic, gastric, and colorectal cancers, its overexpression has been linked to poorer prognosis [61] and resistance to chemotherapy. Its role in other cancers like gallbladder and nasopharyngeal cancers remains unclear [62, 63]. SFN interacts with several signalling pathways, influencing tumour progression and response to treatment. For example, it plays a key role in G2-M checkpoint, by interaction with cyclin-dependent kinases (CDKs). Interaction with proapoptotic proteins and modulation of p53 activity leads to apoptotic inhibition. Furthermore, SFN modulates signalling pathways such as MAPK/ERK, influencing cell survival, proliferation, and migration [64]. Its expression level is proposed as a potential biomarker for diagnosis, prognosis and therapy response. Another marker with the best signature in this cohort, *HSP90AB1*, is a molecular chaperone that stabilises a range of client proteins involved in critical cellular functions. These include oncogenes like EGFR, AKT, and mutant p53, which are key in cell survival, proliferation, and stress responses. HSP90AB1 supports signalling pathways such as PI3K/AKT, MAPK, and NF-kB, which are crucial for tumour progression and resistance to therapy [65]. *HSP90AB1* demonstrated the best area under the curve (AUC) value of 0.82 for distinguishing tumour samples from post-therapy samples, and the best AUC of 0.95 for differentiating high-risk patients from tumour patients (Supplementary Table 2) in our pilot study. Elevated expression of HSP90AB1 has been linked to poor prognosis in various cancers, including lung and breast cancer [66]. Additionally, in the work of Shiraishi et al., HSP90 is highly expressed in 45% of clinical OSCC samples, particularly in cases associated with lymph node metastases [67]. HSP70 and HSP90 are most commonly implicated in the pathogenesis of OSCC. Patient tissue studies have demonstrated that the expression levels of HSP70 and HSP90 increase progressively with the severity of oral epithelial dysplasia (OED) and OSCC [68–70]. HSP90 could therefore also be a potential marker in precancer and offer new possibilities for early diagnosis. Interestingly, HSP90AB1 represents a promising target for inhibition strategies in cancer treatment [65, 71]. The inhibitory effect of novel HSP90 inhibitors on OSCC has already been demonstrated in vitro, as well as in clinical trials [72–74]. A correlation between expression in tissue, smears and response to therapy may indicate that HSP90AB1 may be a suitable prognostic marker for HSP90 inhibitor success and should be considered in further clinical trials. The tumour markers we have identified here have already been linked to several types of cancer. This observation suggests that these markers could be valuable not only for diagnosing head and neck tumours but also for other cancers and with applications across other medical fields. Table 5 provides an overview of these markers across different cancers, analysed at both RNA and protein levels. We found consistent overexpression of these markers in most cancer types, suggesting a potential connection between their expression patterns and tumour development.

**Table 5:** Overview of the regulation of SFN, c-JUN and HSP90AB1 on mRNA and protein levels in different cancer types.

Gene	Protein name	Function	m-RNA	Protein	References
SFN	Stratifin	Adaptor protein, cell cycle regulation; plays a role in the DNA damage response, prevents uncontrolled cell growth.	Ovar up Lung up Colon down	Lung up Cervix up	[75] [76] [93] [94]
HSP90AB1	Heat shock protein AB1	stabilises and folds oncogenic proteins, contributes to tumour progression	Liver up Colon up	Breast up OSCC up Gastro-intestine up	[95] [67] [96] [97] [98]
c-JUN	Jun proto-oncogene	Transcription factor in the AP-1 complex, promotes cell proliferation, apoptosis, differentiation	Breast down Glioblastoma up	Colon up Breast up	[99] [100] [101] [102]

*SNF* and *c-JUN* may act as tumour promoters when overexpressed due to their biological function in oral and oropharyngeal SCC [75, 76]. However, there are also known studies in which SFN acts as a tumour suppressor, e.g. in breast cancer [77]. In contrast, the function of HSP90AB1 in oral and oropharyngeal tumours could be the stabilisation and activation of oncogenes such as *SFN* and *c-JUN* [78] and thus act as an indirect driver, especially in the case of overexpression. The expressions of these markers may even influence each other, which could be verified in further experiments.

It has already been shown in various studies that biomarker panels offer advantages and improved precision in diagnostics compared to individual markers [79, 80]. For example, this was shown by Harlid et al. for early diagnosis of colorectal cancer by DNA methylation signatures [81]. We therefore also carried out combinatorial analyses with the tool CombiROC to increase the precision of our single markers. As described above, the best three combinations are shown in Fig. 4. For Combo VII, only one false positive and one false negative sample were detected in our cohort. However, false negative patients represent a greater challenge in the diagnostics of all cancer types, as they can lead to undiagnosed cases and delay treatment [82]. Combination VII consists of *c-JUN, SFN* and *HSP90AB1*. We also performed combinatorial analysis for differentiating tumour samples from post-therapy patients as well as from high-risk patients. Interestingly, the highest overall AUC values were obtained for the distinction of high-risk patients (Supplementary Table 1).

For better characterisation of the three markers that appear to be the most effective when used together, we further categorised our tumour group based on tumour locations and tumour stages. No significant differences were observed across these subgroups (Supplementary Fig. 6), suggesting that these markers may be independent of factors such as those described above. This indicates that the identified markers could serve as reliable, generalised biomarkers for tumour detection, unaffected by these variables. In this pilot study there are only three HPV positive samples in males, therefore, no comparative statements could be made regarding the marker expression in HPV positive versus HPV negative samples. Another factor that should not be ignored is that the p16 determination is only a surrogate marker for HPV positivity, and therefore it is difficult to make a statement about the actual tumour activity/biology [83, 84]. It would be very interesting to additionally focus on HPV positivity in a larger cohort.

Interestingly, we demonstrated that swabs taken from healthy areas of tumour patients also showed upregulation of *SFN, HSP90AB1*, and *c-JUN* (Supplementary Fig. 7). When looking at cytological preparations of these samples, it is noticeable that they are more similar to the smears of the tumour areas. Dysplastic / atypically altered cells can also be found. One possible explanation for this could be the NaCl mouthwash solution prior to the swab collection. This could have led to the displacement of tumour cells and contamination of the healthy swabs with modified cells, thus resulting in higher expression levels of these markers. Another biological process that should be considered here is field cancerization. In this process, large areas of cells on a tissue surface or within an organ can be affected by carcinogenic changes [85]. The effect of field cancerization proposes that normal tissue adjacent to the primary tumour harbours pre-neoplastic alterations [86]. Alternatively, it is also conceivable that the genes we observed to be upregulated in tumour patients might be induced by the TME signalling transmitted into healthy areas, potentially stimulating increased transcription. Signalling molecules could be transmitted through immune cell or immune-mediating molecules via the saliva, affecting the entire region, which could result in upregulation of the marker. The previously described phenomenon appears to be limited to the mRNA level, as immunofluorescence staining of healthy areas in tumour patients showed no protein expression, suggesting that this effect may be limited to mRNA regulation. Further studies are needed to validate the biomarkers analysed in this work for routine clinical use. Factors e.g. HPV positivity and sex specificity, should be evaluated in larger cohorts. Given the simplicity of the swab test, these markers could be integrated into daily practice via a clinical study, enabling additional data collection and assessment of their patient benefit through this non-invasive approach.

To this end, the approach should be tested in various clinical settings, as it would also be advantageous to carry out such tests not only in clinics but also in ENT practices and dental ambulances. Another important aspect is early diagnosis. It would be very interesting to analyse the expression of the potential biomarkers in precancerous lesions (SIN/CIS), as this study unfortunately does not allow us to make any statement about the expression in such areas or precancerous lesions. Various studies have already identified differences between precancer (oral leukoplasia/dysplasia) and cancer or even possible predictions of malignant degeneration [87–89]. If these patients with precancer lesions showed similar expression values to the tumour patients, such a combinatorial test could not only support the diagnosis but also be used as a screening variant. Consequently, identifying molecular biomarkers that can distinguish lesions with a higher risk of progression is crucial.

### Protein and mRNA expression of biomarker in tissue

As shown representatively in Fig. 5, protein-level analysis via antibody staining confirmed the tumour specificity of the markers. The images demonstrate oropharyngeal tumours with strong expression of the proteins within the tumour, while showing little to no expression in healthy tissue. SFN and HSP90AB1 displayed strong cytoplasmic protein expression, while c-JUN exhibited both cytoplasmic and nuclear staining. The increased expression of SFN is consistent with the observation of SFN expression in cervical cancer [76]. Additionally, Fig. 5d shows the upregulation of these markers at the mRNA level in fresh tissue of tumour patients. These results correlate with the measurements obtained from the tumour swabs and the protein expression. *HSP90AB1* expression was not only detected in the cytoplasm of tumour cells, but could also be detected in immune cells in tumours heavily infiltrated by lymphocytes (Supplementary Fig. 8). It is known that HSP90AB1 is expressed in a variety of immune cells, including lymphocytes, monocytes, and macrophages. This chaperone protein is crucial for stabilising and folding other proteins, which is particularly important in immune cells due to their dynamic response to stress and pathogenic threats [90, 91]. In addition, we verified our results obtained for the top three biomarkers in tissue using the online tool OncoDB, which accesses RNA-seq data from both the TCGA study and the GTEx study [92]. Compared to our results, the difference between normal and tumour tissue was smaller, but similar trends could be observed, with the greatest difference derived for the databank for the biomarker HSP90AB1 (Supplementary Fig. 9). It is noteworthy that these are RNA sequencing data, whereas in our study, qPCR data are shown. Above all, the focus of our study lay on oral/oropharyngeal localisations; the databank comprised all HNSC areas. Verification of gender specificity using OncoDB revealed no significant differences, which could also be due to the different locations (all HNSC subtypes) or to the larger sample size of males (*n* = 375) and females (*n* = 132). When looking at the T classification (T stages), no significant differences were found for all three biomarkers during database verification. This is consistent with the trend of a possibly stage-independent expression of the markers in our study, which we show in Supplementary Fig. 6.

### Limitations

This study is a pilot study on diagnostic accuracy with an initially limited number of samples. To improve and further validate this study, this approach should be repeated in the future with a significantly larger sample size and gender-specific data, which could be used to develop a predictive model through further validation. In addition, factors such as HPV positivity, etc., should be further analysed and additional samples obtained. We tested the mRNA-based biomarkers in individuals who had confirmed SCC or who were classified as high-risk (but did not have SCC). At this time, we cannot comment on the expression of the presented markers in the precancerous stage. A limiting aspect of this study could also be the upregulation of markers in clinically healthy patients and healthy mucosa of tumour patients, which is why a combination of markers is of great importance. Nevertheless, promising results have been achieved that have the potential to support the diagnosis of oral and oropharyngeal SCC using molecular biological methods in the future.

## Conclusion

In summary, we were able to identify *c-JUN, SFN* and *HSP90AB1* as potential mRNA-based markers for the diagnosis of oral and oropharyngeal SCC in oral smears of a small cohort by RNA sequencing and verified them in a wider cohort via RT-qPCR. The combination of these markers led to a profound increase in diagnostic precision. Nonetheless, these results should be confirmed in a larger, more heterogeneous cohort. Notably, the biomarkers assessed demonstrated greater diagnostic utility in male participants. Immunofluorescence staining demonstrates their tumour specificity in FFPE tumour tissue. Interestingly, the identified markers are already associated with therapeutic approaches for cancer therapy and could therefore be helpful in improving not only diagnostics or therapy monitoring.

## Supplementary information

Supplementary Figures

## Data Availability

The datasets generated and analysed during the present study are available from the corresponding author upon reasonable request.
