# Supplementary material for: Diagnostic accuracy of combinatorial mRNA biomarkers for non-invasive detection and therapy monitoring of oral and oropharyngeal SCC
Source: Br J Cancer. 2026 Jan 8;134(6):961–74. doi: 10.1038/s41416-025-03313-w (PMC12960811; doi:10.1038/s41416-025-03313-w)
Supplement: Supplementary file 1 — Supplementary Figures [file 41416_2025_3313_MOESM1_ESM.docx]

**
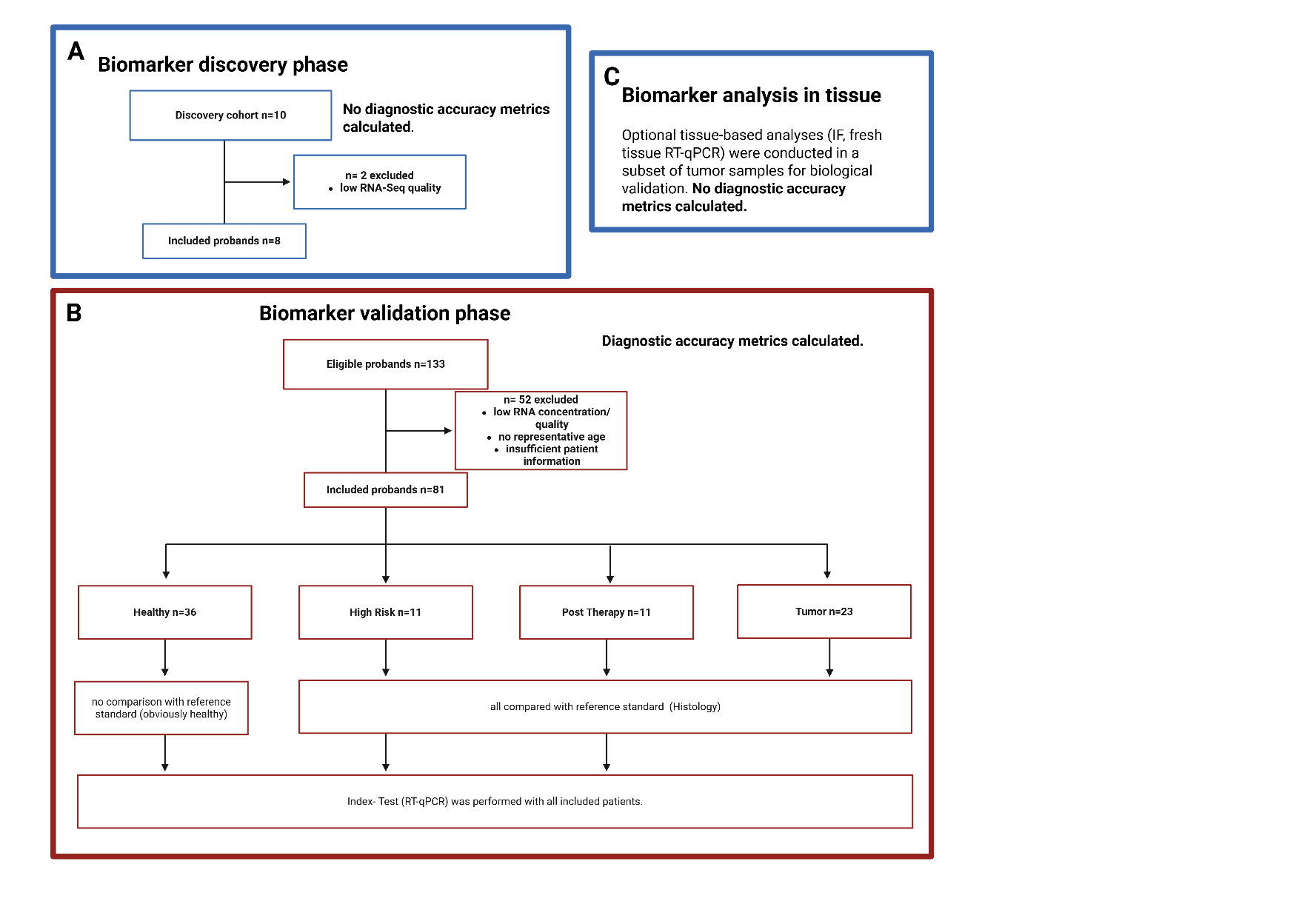
Supplementary Figures**

***Suppl.Fig.1) Flow-Chart of calculated diagnostic accuracy metrics.*** *The flowchart shows the different parts of the study:* ***A)*** *Biomarker discovery phase,* ***B)*** *Biomarker validation phase,* ***C)*** *Biomarker analysis in tissue. The diagnostic metrics were analyzed in part B and are shown transparently in the flowchart accordingly. The data presented refer to the analyses of diagnostic accuracy in men and women.*


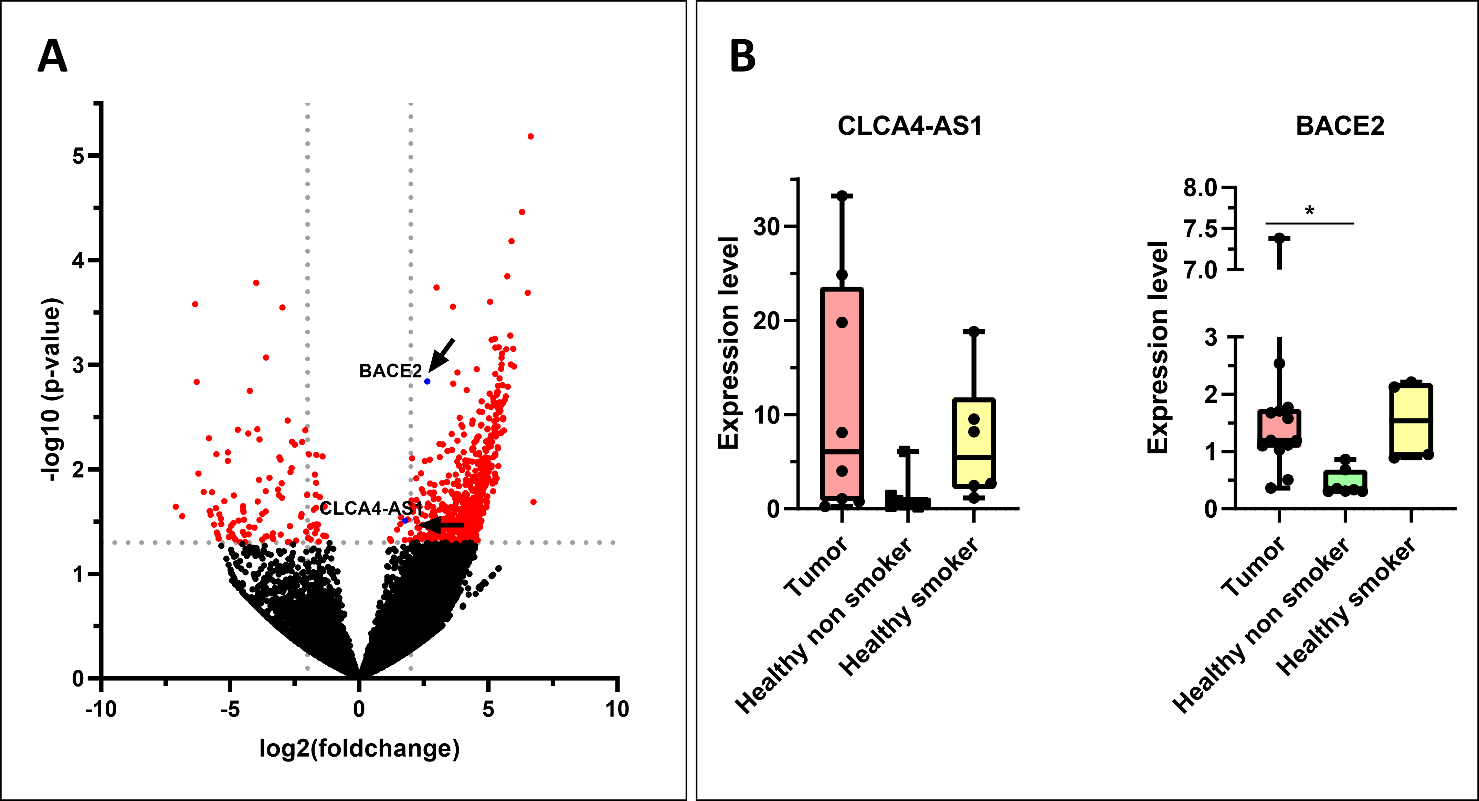


***Suppl.Fig.2) Smoking behavior influences gene regulation.*** *Sequencing data are given in a* *Volcano plot in* ***A),*** *which shows the differential gene expression between smoking tumor patients and non-smoking healthy probands. Blue highlighted genes BACE2 and CLC4A-AS1 are verified with RT-qPCR* ***B)*** *and show upregulation in tumor as well as healthy smoker, compared to healthy non-smoker. (Welch's t-test, two-tailed, 95 % confidence interval, * p<0.05, ** p < 0.01).*

.

**
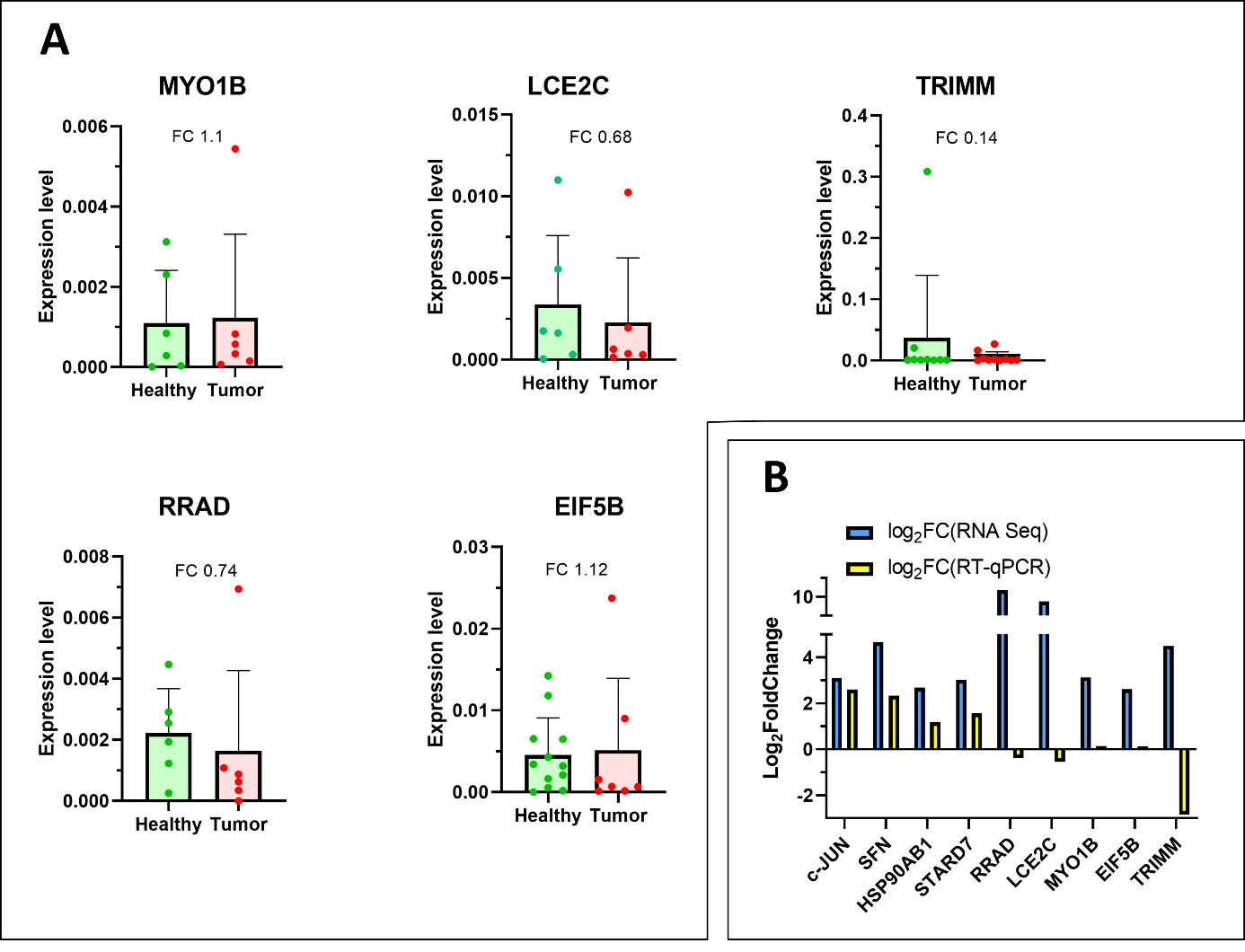
**

***Suppl.Fig. 3) Sequencing data differ from qPCR verification in a wider cohort. A)*** *Bar plots of gene expression analysis with RT-qPCR for the groups healthy and tumor shows, that the sequencing data* *it is transferable to a limited extent to a bigger cohort. Representative for this are the genes MYO1B, LCE2C, TRIMM, RRAD, and EIF5B. (Welch's t-test, two-tailed, 95 % confidence interval, * p<0.05, ** p < 0.01).* ***B)*** *Comparison of log2FC from RNA-Seq and RT-qPCR.*

***Suppl.Table 1) Overview of ROC analyses of c-JUN, SFN, HSP90AB1, STARD7.*** *Group healthy probands, tumor patients after treatment und high risk patients were analyzed against the tumor group. In this table all AUC, SE and SP values for the genes c-JUN, SFN, HSP90AB1 and STARD7 are given.*

|  | **Healthy** | | **After Treatment** | | **High Risk** | |
| --- | --- | --- | --- | --- | --- | --- |
| **c-JUN** | **AUC** | **SE / SP** | **AUC** | **SE / SP** | **AUC** | **SE / SP** |
| **Tumor** | 0.88 | 0.923/0.769 | 0.671 | 0.923/0.636 | 0.89 | 0.923/0.818 |
| **SFN** |  |  |  |  |  |  |
| **Tumor** | 0.87 | 0.923/0.769 | 0.706 | 0.846/0.727 | 0.83 | 0.629/0.909 |
| **HSP90AB01** |  |  |  |  |  |  |
| **Tumor** | 0.75 | 0.615/0.846 | 0.783 | 1.00/0.636 | 0.95 | 0.769/1.00 |
| **STARD7** |  |  |  |  |  |  |
| **Tumor** | 0.64 | 0.462/0.923 | 0.717 | 0.846/0.545 | 0.67 | 0.846/0.545 |

***Suppl.Table 2) Overview of ROC analyses of all combinations.*** *Group healthy probands, tumor patients after treatment und high risk patients were analyzed against the tumor group. In this table all AUC, SE and SP values for the genes c-JUN, SFN, HSP90AB1 and STARD7 are given.*

|  | **Healthy** | | **After Treatment** | | **High Risk** | |
| --- | --- | --- | --- | --- | --- | --- |
| **Combo I c-JUN+ SFN**  **c-JUN+ SFN** |  |  |  |  |  |  |
| **Tumor** | 0.89 | 0.923/0.846 | 0.713 | 0.796/0.727 | 0.902 | 0.923/0.818 |
| **Combo II**  **c-JUN+HSP90AB1** |  |  |  |  |  |  |
| **Tumor** | 0.88 | 0.923/0.769 | 0.783 | 1.0/0.636 | 0.951 | 0.846/1.0 |
| **Combo III**  **c-JUN+STARD7** |  |  |  |  |  |  |
| **Tumor** | 0.89 | 0.923/0.846 | 0.692 | 0.615/0.818 | 0.972 | 0.923/1.0 |
| **Combo IV**  **SFN+HSP90AB1** |  |  |  |  |  |  |
| **Tumor** | 0.86 | 0.846/0.846 | 0.797 | 0.923/0.727 | 0.951 | 0.769/1.0 |
| **Combo V**  **SFN+STARD7** |  |  |  |  |  |  |
| **Tumor** | 0.88 | 0.769/0.923 | 0.706 | 0.615/0.818 | 0.937 | 0.923/0.909 |
| **Combo VI**  **HSP90AB1+STARD7** |  |  |  |  |  |  |
| **Tumor** | 0.74 | 0.692/0.769 | 0.72 | 0.923/0.545 | 1 | 1.0/1.0 |
| **Combo VII**  **c-JUN+SFN+STARD7** |  |  |  |  |  |  |
| **Tumor** | 0.91 | 0.923/0.923 | 0.734 | 0.841/0.727 | 0.958 | 0.846/1.0 |
| **Combo VIII**  **c-JUN+SFN+STARD7** |  |  |  |  |  |  |
| **Tumor** | 0.9 | 0.923/0.846 | 0.713 | 0.815/0.818 | 0.972 | 0.923/1.0 |
| **Combo IX**  **c-JUN+HSP90AB1+STARD7** |  |  |  |  |  |  |
| **Tumor** | 0.89 | 0.923/0.846 | 0.664 | 0.426/0.909 | 1 | 1.0/1.0 |
| **Combo X**  **SFN+HSP90AB1+STARD7** |  |  |  |  |  |  |
| **Tumor** | 0.89 | 0.923/0.769 | 0.692 | 0.538/0.909 | 1 | 1.0/1.0 |
| **Combo XI**  **c-JUN+SFN+HSP90AB1+STARD7** |  |  |  |  |  |  |
| **Tumor** | 0.89 | 0.923/0.846 | 0.692 | 0.538/0.909 | 1 | 1.0/1.0 |

***Suppl.Table 3) Overview optimal cut-off values of all single marker.*** *Group healthy probands, tumor patients after treatment und high risk patients were analyzed against the tumor group. In this table optimal cut-off values for the genes c-JUN, SFN, HSP90AB1 and STARD7 are given.*

| **optimal Cut-off** | **Healthy** | **After Treatment** | **High Risk** |
| --- | --- | --- | --- |
| **c-JUN** | **0.354** | **0.519** | **0.371** |
| **SFN** | **0.566** | **0.509** | **0.629** |
| **HSP90AB1** | **0.277** | **0.489** | **0.718** |
| **STARD7** | **0.586** | **0.408** | **0.456** |


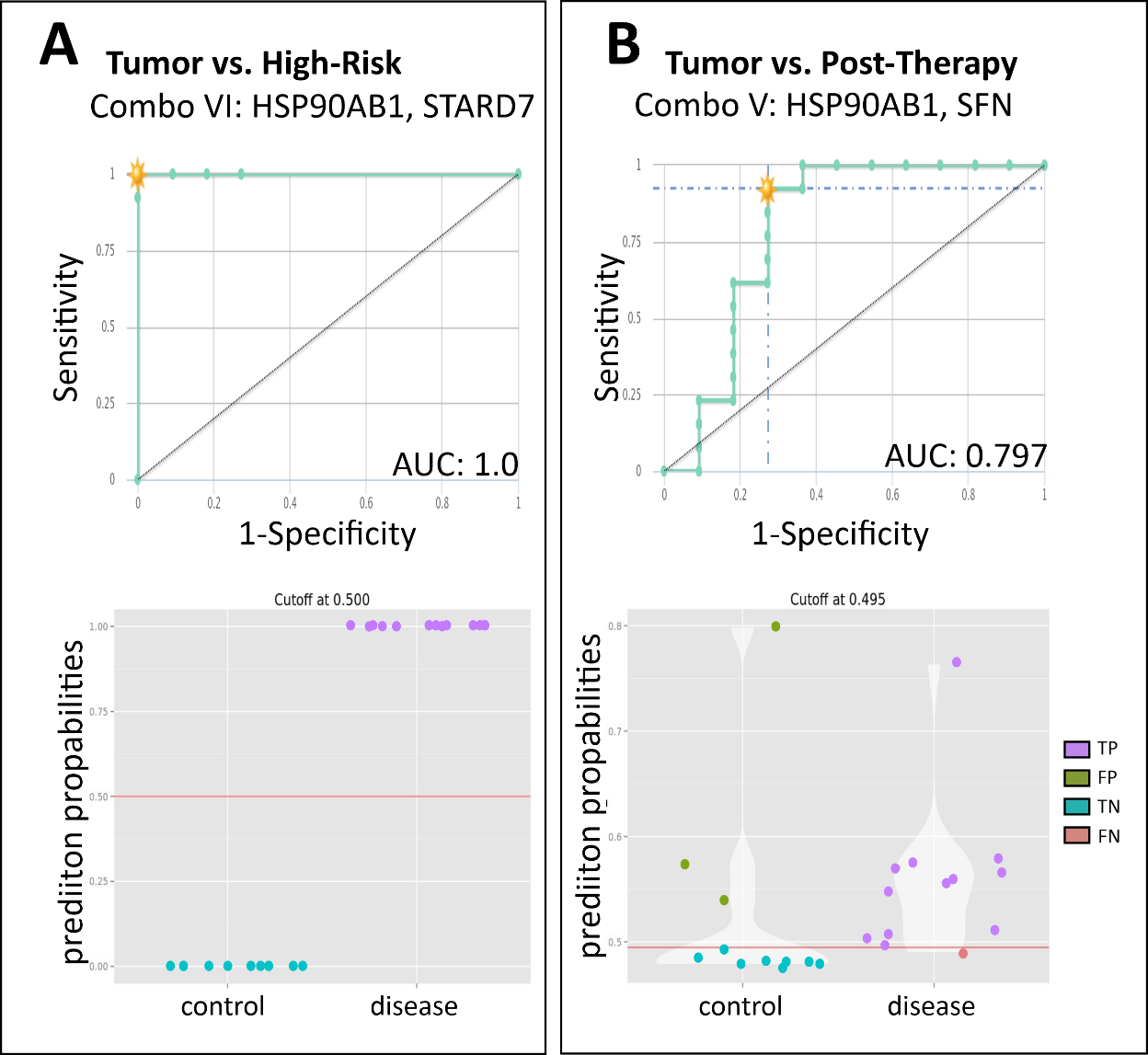


***Suppl.Fig.4) Combinatorial analysis of RT-qPCR data results. A)*** *Diagnostic accuracy of combination VI with corresponding ROC analysis (AUC:1.0) of Tumor vs. High-Risk group.* ***B)*** *Diagnostic accuracy of combination V with corresponding ROC analysis (AUC:0.797) of Tumor vs. Post-Therapy group.*


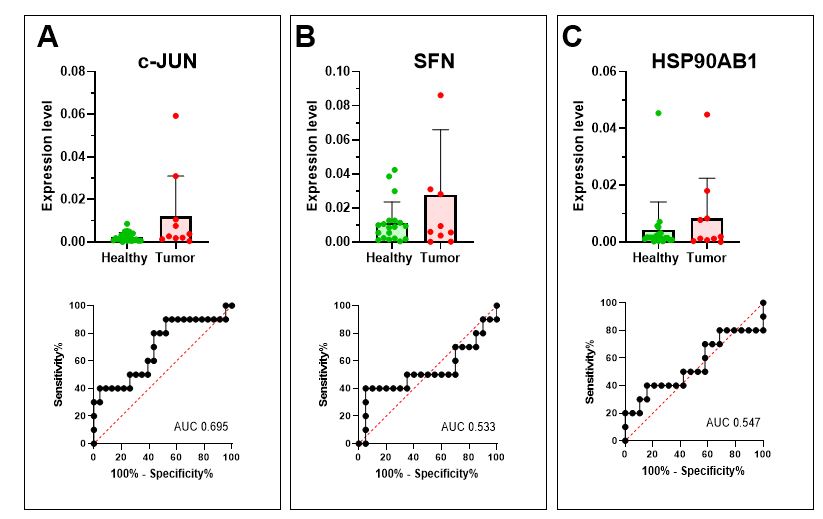


***Suppl.Fig.5) qPCR verification of biomarkers in women.***  *The barplots show expression levels of the biomarker* ***A)*** *c-JUN,* ***B)*** *SFN and* ***C)*** *HSP90AB1 in female tumor patients compared to female healthy probands (Welch's t-test, two-tailed, 95 % confidence interval, * p<0.05, ** p < 0.01). No significant differences between healthy probands and tumor patients could be determined. ROC Analysis with corresponding AUC values are given.*


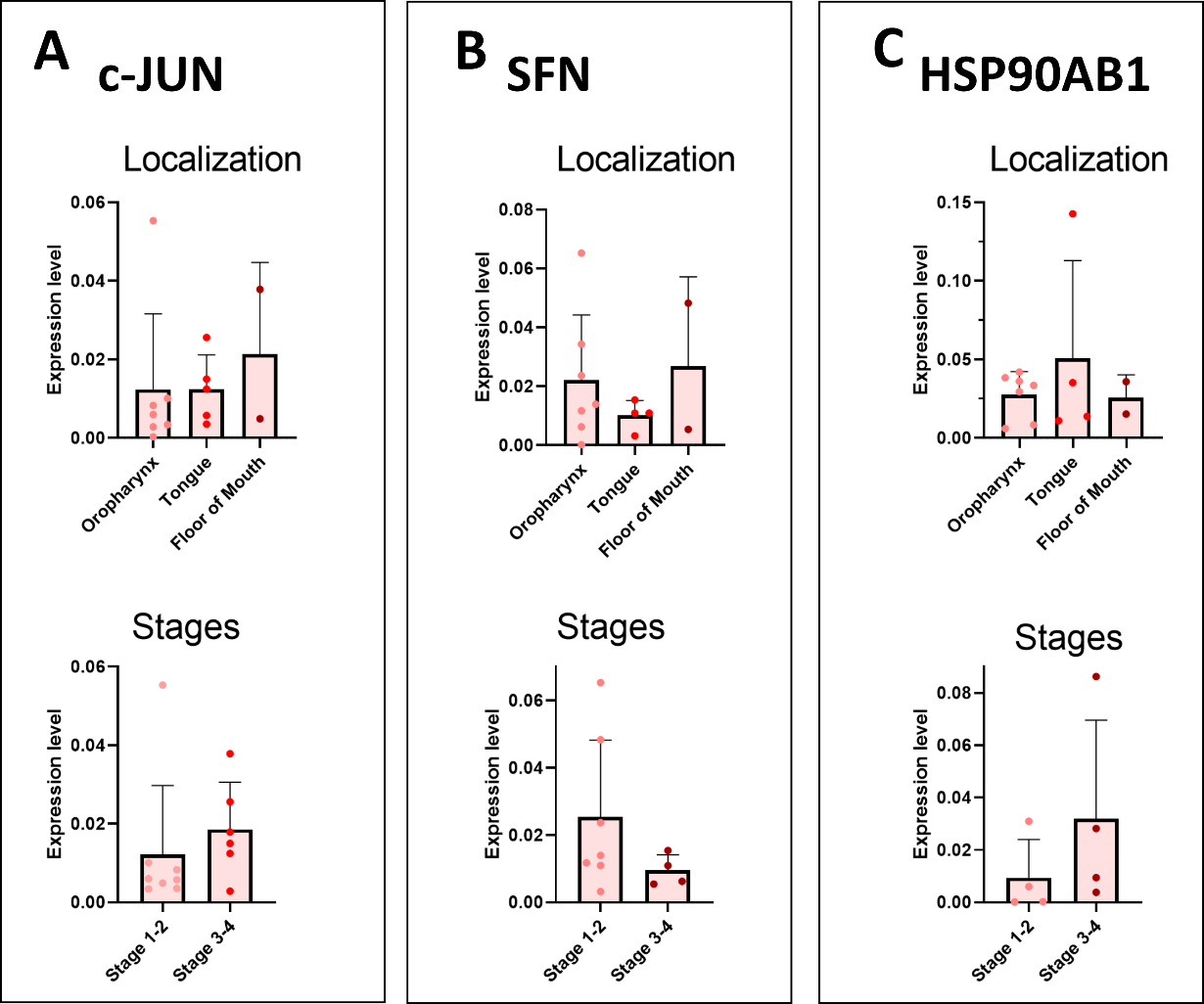


***Suppl.Fig.6) Characterization of biomarkers shows independence from localization and T classification.*** *The bar plots show expression levels of* ***A)*** *c-JUN,* ***B)*** *SFN and* ***C)*** *HSP90AB1 in male tumor patients. Different tumor localizations and stages (TNM classification) are compared with each other. No significant differences could be measured for all three criteria. Therefore, marker c-JUN, SFN and HSP90AB01 can be considered independent of these classifications (Welch's t-test, two-tailed, 95 % confidence interval, *p<0.05, ** p < 0.01).*


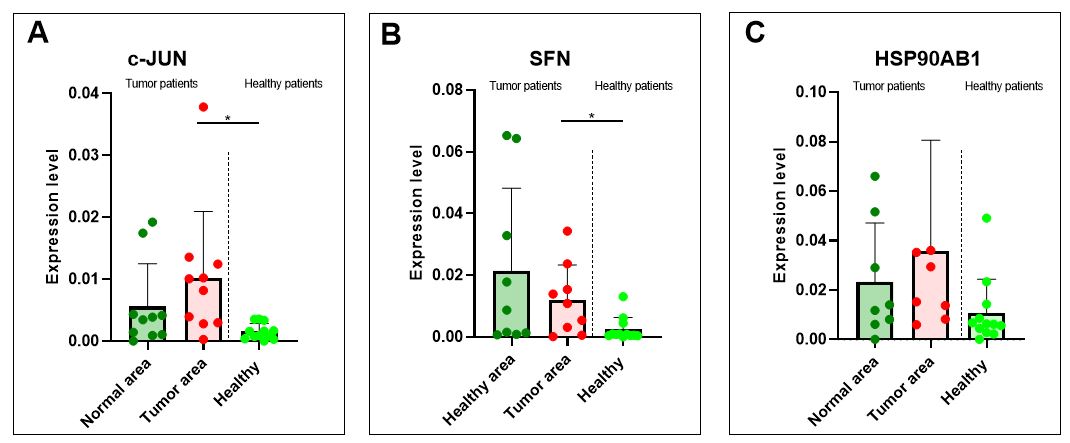


**
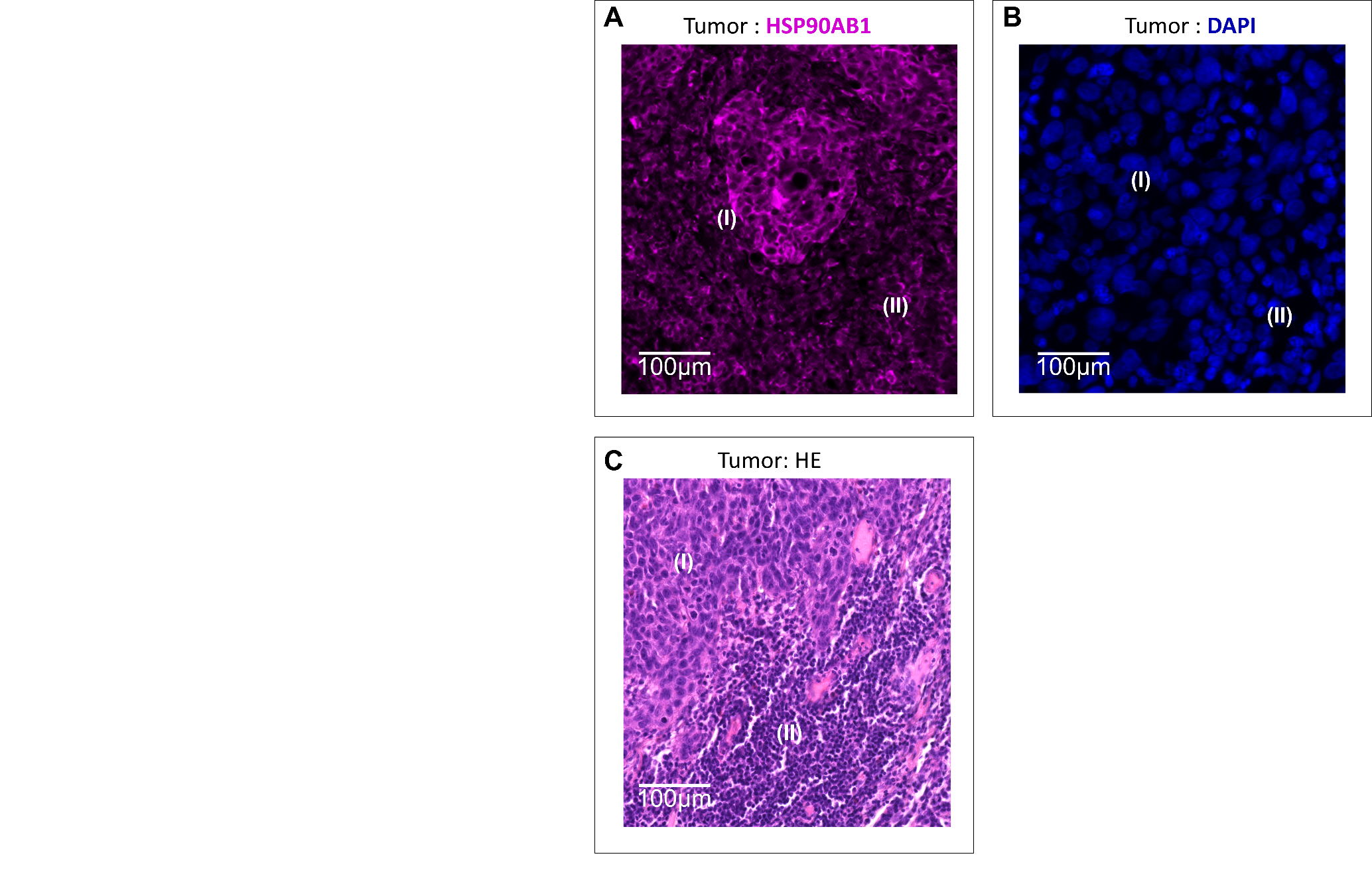
**

***Suppl.Fig.7) Normal mucosa of tumor patients shows upregulation of potential markers in comparison to the healthy group****. Higher expressions of c-JUN, SFN and HSP90AB1 is measured in the healthy mucosa of tumor patients, in comparison to the expression of healthy probands.* *In some cases, even higher expressions were measured in the healthy mucosa of tumor patients than in the tumor areas (Welch's t-test, two-tailed, 95 % confidence interval, * p<0.05, ** p < 0.01).*

***Suppl.Fig.8) HSP90AB1 is expressed in tumor and infiltrating immune cells****. Representative image of lymphocyte infiltrated tumor cell cluster.* ***A)*** *HSP90AB1 staining shows expression in tumor cell cluster (I) also Lymphocytes show cytoplasmic HSP90AB1 expression (II).* ***B)*** *DAPI filter shows nuclear staining (I) tumor, (II) lymphocytes.* ***C)*** *Representative HE-image of infiltration of lymphocytes (II), tumor (I). Scale bar = 100 µm.*


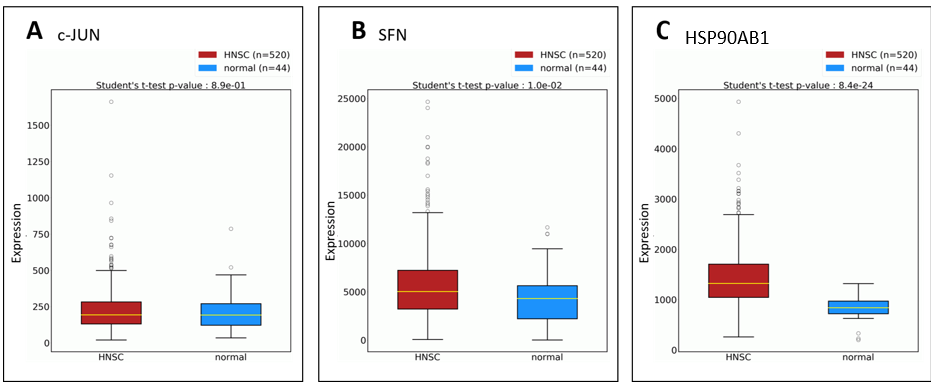


***Suppl.Fig.9) Biomarker verification with OncoDB shows upregulation in HNSC samples.*** *Expression data visualization reproduced from OncoDB (*[*https://oncodb.org*](https://oncodb.org)*).
The figure was generated using publicly available data in OncoDB and is reproduced here for illustrative purposes.* *RNA-seq data from HNSC tissue samples (n=520) from the TCGA study are plotted against healthy tissue samples (n=44) from the GTEx study. All three markers show upregulation in tumor samples, with c-JUN having the lowest log_2_FC (0.01) and HSP90AB1 having the highest log_2_FC (0.66).* *The log_2_FC of SFN is 0.28. (Student`s t-test, two-tailed, 95 % confidence interval, * p<0.05, ** p < 0.01*).
